# Supplementary material for: US adults with diabetes mellitus: Variability in oral healthcare utilization
Source: PLoS One. 2021 May 5;16(5):e0251120. doi: 10.1371/journal.pone.0251120 (PMC8099075; doi:10.1371/journal.pone.0251120)
Supplement: S1 Table — (DOCX) [file pone.0251120.s001.docx]

**S1 Table. Dental visit compliance by urban and rural participants’ characteristics (n=40,585; N=19,281,982).**

| Characteristics | Adjusted OR (95% CI)* | |
| --- | --- | --- |
|  | Urban | Rural |
| Age (years)  18-44 vs 65+  45-64 vs 65+ | 1.10 (0.88-1.38)  1.03 (0.89-1.19) | 1.00 (0.64-1.59)  0.94 (0.65-1.35) |
| Sex  Female vs Male | 1.19 (1.06-1.34) | 1.25 (0.98-1.59) |
| Race/Ethnicity  Non-Hispanic (NH) Black vs NH White  Hispanic vs NH White  NH Other/Multiracial vs NH White | 0.92 (0.80-1.06)  0.90 (0.74-1.10)  1.05 (0.81-1.36) | 1.09 (0.79-1.50)  0.82 (0.48-1.40)  1.49 (1.03-2.16) |
| Marital status  Divorced/Separated/Never married vs Couple  Widowed vs Couple | 0.95 (0.83-1.09)  0.86 (0.74-1.00) | 0.64 (0.49-0.84)  0.96 (0.68-1.36) |
| Education  High school (HS) graduate vs < HS  Some college/Technical vs < HS  College graduate vs < HS | 1.11 (0.93-1.33)  1.30 (1.08-1.56)  1.81 (1.48-2.21) | 1.16 (0.79-1.70)  1.79 (1.15-2.79)  2.46 (1.58-3.84) |
| Income ($)  15,000-34,999 vs <15,000  35,000-49,999 vs <15,000  50,000+ vs <15,000 | 1.10 (0.92-1.31)  1.37 (1.12-1.68)  2.27 (1.83-2.82) | 0.76 (0.56-1.04)  0.72 (0.47-1.11)  1.30 (0.87-1.95) |
| Employment  Homemaker/Student/Retired vs Employed  Not employed/ Unable to work vs Employed | 1.22 (1.05-1.42)  1.00 (0.85-1.18) | 0.98 (0.71-1.35)  0.86 (0.61-1.21) |
| Healthcare coverage^†^  Yes vs No | 1.63 (1.29-2.06) | 2.44 (1.43-4.16) |
| Personal doctor^‡^  Yes vs No | 1.41 (1.14-1.75) | 1.50 (0.94-2.40) |
| Medical cost^§^  Yes vs No | 0.68 (0.57-0.81) | 0.76 (0.57-1.02) |
| Self-reported general health  Good/Better vs Fair/Poor | 1.21 (1.08-1.36) | 1.13 (0.87-1.46) |
| Number of permanent teeth removed  1-5 vs None  6+ vs None  All vs None | 1.02 (0.90-1.16)  0.82 (0.71-0.95)  0.27 (0.22-0.33) | 1.23 (0.88-1.72)  0.98 (0.71-1.36)  0.25 (0.17-0.37) |
| Smoking status^ǁ^  Current vs Never  Former vs Never | 0.70 (0.59-0.83)  0.90 (0.80-1.01) | 0.70 (0.51-0.96)  1.27 (0.98-1.64) |
| Average alcohol intake in past 30 days  2+ drinks/day vs None  1 drink/day vs None | 0.96 (0.83-1.11)  1.12 (0.98-1.28) | 1.00 (0.71-1.40)  1.36 (1.03-1.80) |
| Poor mental health in past 30 days  Yes vs No | 0.94 (0.84-1.06) | 0.83 (0.61-1.11) |
| Poor physical health in past 30 days  Yes vs No | 0.98 (0.87-1.10) | 0.78 (0.58-1.05) |
| Exercise in past 30 days^¶^  Yes vs No | 1.26 (1.12-1.42) | 1.32 (1.04-1.67) |
| Body Mass Index (BMI)^#^  Underweight/Normal weight vs Obese  Overweight vs Obese | 1.21 (1.02-1.43)  1.18 (1.05-1.33) | 0.69 (0.47-1.01)  0.80 (0.62-1.03) |

^*^Odds ratios (OR) and 95% CI are adjusted for all the variables listed in the column and the complex sampling design.

^†^Healthcare coverage: Has any kind of health care coverage, including health insurance, prepaid plans such as health maintenance organization (HMO), or government plans such as Medicare, or Indian Health Service.

^╪^Personal doctor: Has a personal doctor or health care provider.

^§^Medical cost: Could not see a doctor because of cost

^ǁ^Smoking status: Current: smokes every day or some days, Former: smoked before, Never: never smoked.

^¶^Exercise: Participated in any physical activities or exercises such as running, calisthenics, golf, gardening, or walking for exercise in the past 30 days.

^#^BMI; Underweight/Normal weight: BMI<25, Overweight: 25≤BMI <30, Obese: BMI≥30
